# Interstitial Granulomatous Dermatitis as a Rare Paraneoplastic Manifestation of Non-small Cell Lung Cancer: A Case Report

**DOI:** 10.7759/cureus.90056

**Published:** 2025-08-14

**Authors:** Maryam Abdurrahman, Zia Kanji

**Affiliations:** 1 Internal Medicine, Milton Keynes University Hospital, Milton Keynes, GBR

**Keywords:** granulomatous dermatitis, interstitial granulomatous dermatitis, lung cancer, non small cell lung carcinoma, paraneoplastic dermatoses, pet avid lung nodule, skin biopsy, squamous cell carcinoma

## Abstract

Interstitial granulomatous dermatitis (IGD) is an uncommon inflammatory skin disorder, characterised histologically by mid‑dermal non‑caseating granulomas. While often linked to autoimmune diseases and drug reactions, its occurrence as a paraneoplastic phenomenon is rare.

We report a case of a 69‑year‑old male with a two‑year history of a progressive erythematous maculopapular rash involving the trunk and extremities, associated with fatigue, weight loss, and myalgia. Initial superficial biopsy suggested granuloma annulare, but a deeper biopsy confirmed IGD, with a preserved Grentz zone and multinucleate giant cells. Laboratory tests revealed elevated ESR, as well as CRP, hypercalcaemia, and IgM paraproteinaemia. Imaging identified a PET‑avid, cavitating left lower lobe lung nodule. CT‑guided biopsy was non‑diagnostic, prompting wedge resection. Histology confirmed non‑small cell lung cancer, squamous subtype. Notably, the rash markedly improved following lung resection. IGD can be a cutaneous marker of occult malignancy. Persistent granulomatous skin eruptions unresponsive to standard therapies should prompt consideration of systemic disease, including neoplasia. This case underscores the value of a multidisciplinary diagnostic approach and highlights the potential for symptom resolution with targeted treatment of the underlying tumour.

## Introduction

Interstitial granulomatous dermatitis (IGD) is an inflammatory skin disorder with a distinctive histopathological profile, often presenting clinically as erythematous plaques, nodules, or linear lesions [1,2]. Hallmark histological features include mid-dermal non-caseating granulomas with multinucleate giant cells and a preserved Grentz zone, distinguishing it from other granulomatous dermatoses such as sarcoidosis or granuloma annulare [3].

Although uncommon, IGD has been reported as a paraneoplastic manifestation, arising in the context of underlying malignancy or other chronic inflammatory drivers [3-5]. Paraneoplastic presentations are exceptionally rare; a literature review identified only 37 reported cases of paraneoplastic IGD/palisaded neutrophilic and granulomatous dermatitis (PNGD), with haematological malignancies accounting for roughly one quarter, and a smaller proportion linked to solid organ tumours, including lung carcinoma [6,7]. These reports highlight the importance of evaluating chronic granulomatous skin eruptions as potential markers of occult malignancies [5]. Recognition of cutaneous paraneoplastic syndromes can precede the diagnosis of internal malignancies, enabling earlier detection and potentially improving prognosis [6].

Early recognition and management of the underlying neoplasm can often result in significant improvement or resolution of cutaneous symptoms [3,4,7]. This case adds to the limited evidence base, describing a patient with histologically confirmed IGD occurring in association with a PET-avid, cavitating lung nodule, later diagnosed as squamous cell carcinoma of the lung [8]. Association with pulmonary squamous cell carcinoma is particularly uncommon, with only a single published case describing complete resolution of IGD and associated arthritis following surgical resection of a bronchial squamous cell carcinoma [9].

## Case presentation

**Figure 1:**
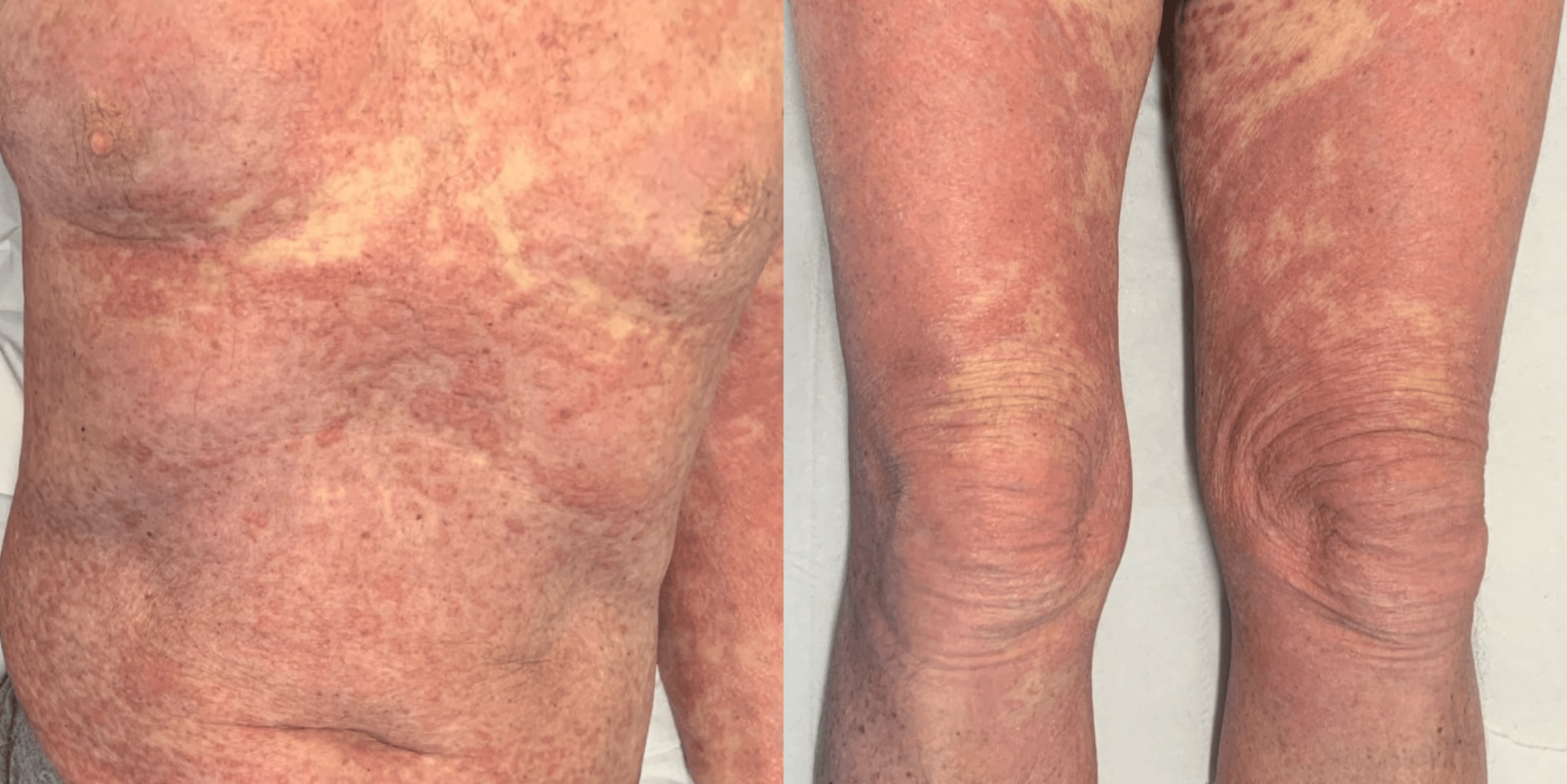
Widespread erythematous maculopapular rash at the time of presentation.

A 69-year-old retired chartered surveyor presented with a two-year history of a progressive, widespread, erythematous maculopapular rash involving the trunk and extremities, covering 40%-50% of the total body surface area (Figure 1). The eruption began in 2022, following travel to Cambodia, as a blistering rash on the lower limbs, which later spread to the upper limbs and was diagnosed in primary care as pityriasis rubra pilaris (PRP). The rash was first mentioned during a rheumatology follow-up appointment for polymyalgia rheumatica (PMR) in September 2023. It worsened after cessation of corticosteroids prescribed for PMR and was associated with fatigue, myalgia, and a six-kg weight loss. He has a 45-pack-year smoking history (now an ex-smoker). His past medical history includes osteoporosis, hypertension, gout, mild chronic obstructive pulmonary disease (COPD), and PMR.

**Figure 2:**
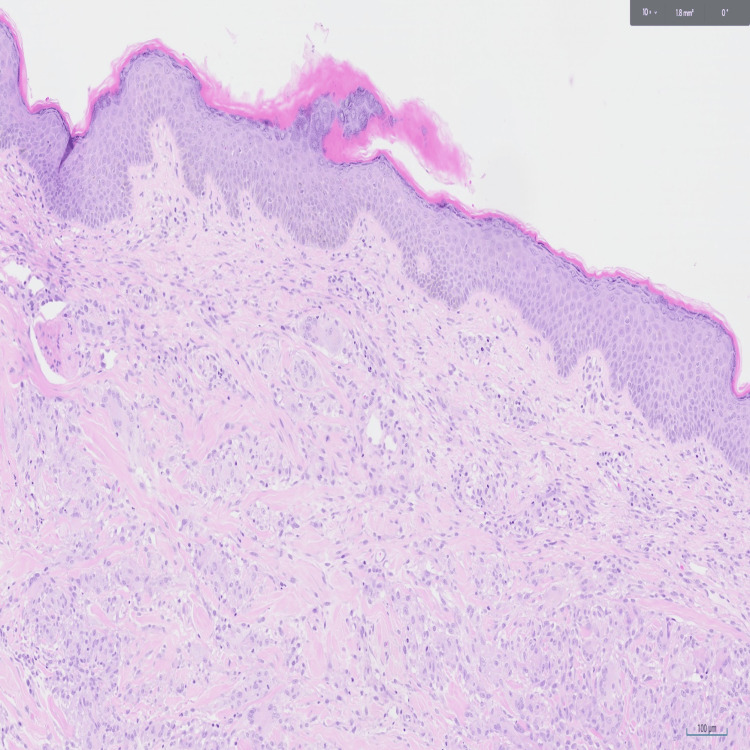
Histology (H&E stain) showing mid‑dermal granulomas with multinucleated giant cells and a preserved Grenz zone.

A punch biopsy performed in primary care in August 2024 favoured a diagnosis of granuloma annulare, and he was started on topical steroids with negative direct immunofluorescence studies. Despite this, the rash continued to progress, becoming more extensive and refractory to topical treatments, prompting further evaluation by the dermatology team. In October 2024, a deep incisional biopsy from the upper right back revealed mid-dermal non-caseating granulomas with multinucleate giant cells and a preserved Grenz zone (Figure 2). Histopathological examination showed no evidence of connective tissue necrosis or degeneration, and additional stains for atypical bacteria and fungi were negative. These findings confirmed the diagnosis of IGD.

A biopsy of the right gluteal region for direct immunofluorescence remained negative, ruling out autoimmune connective tissue diseases such as lupus erythematosus or dermatomyositis. Antineutrophil cytoplasmic antibody (ANCA) and antinuclear antibody (ANA) screens were negative, excluding vasculitis and systemic lupus erythematosus (SLE). Serum free light chains were negative, excluding multiple myeloma. ESR and CRP were persistently elevated, indicating ongoing systemic inflammation. As part of dermatological management, the patient was prescribed clobetasol shampoo, calcipotriol foam and greasy emollients for regular use. Despite these measures, the rash persisted.

He was found to have lingering anaemia and had been reviewed by Haematology, Rheumatology, and Gastroenterology over 2023. Blood tests showed a mild normocytic anaemia, ESR >140, CRP up to 86, elevated ferritin, and low transferrin saturations, consistent with functional iron deficiency. Endoscopy demonstrated mild gastropathy, but biopsies were normal.

**Figure 3:**
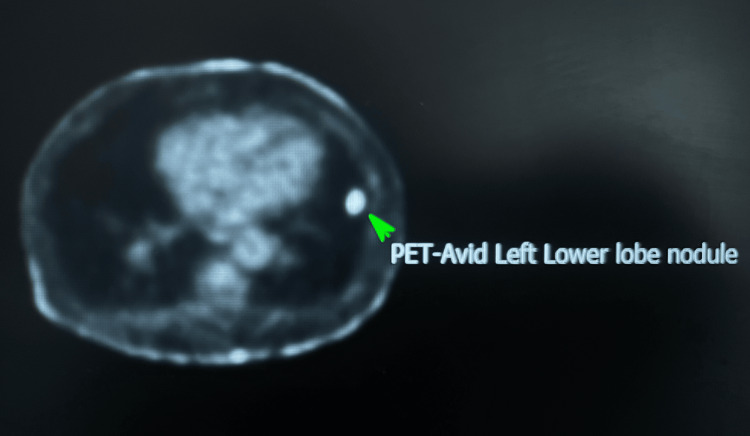
Intensely FDG-avid left lower lobe lung nodule measuring 15 mm (SUVmax 5.8), consistent with bronchogenic malignancy (stage T1b N0 M0). FDG, Fluorodeoxyglucose

In October 2024, he was admitted for management of hypercalcaemia (serum calcium 3.12 mmol/L). Beta-2 microglobulin, parathyroid hormone (PTH), angiotensin-converting enzyme (ACE), and myeloma screen were negative. A CT scan of the thorax, abdomen, and pelvis performed during this admission revealed a suspicious cavitating lesion in the lower lobe of the left lung, with severe background emphysema. In November 2024, PET-CT confirmed an avid cavitating nodule in the left lower lobe of the lung (Figure 3), staged radiologically as T1b N0 M0. The case was discussed at the respiratory multidisciplinary team meeting, and the decision was made to proceed with CT-guided biopsy.

In December 2024, the CT-guided biopsy was non-diagnostic due to insufficient tissue. Following discussion at the respiratory multidisciplinary team meeting, it was decided that, given the high PET avidity, lack of diagnostic clarity, and suspicion that IGD was likely paraneoplastic in origin and associated with the PET-avid lung nodule, the best course of action was to proceed with surgical wedge resection and intraoperative frozen section.

**Figure 4:**
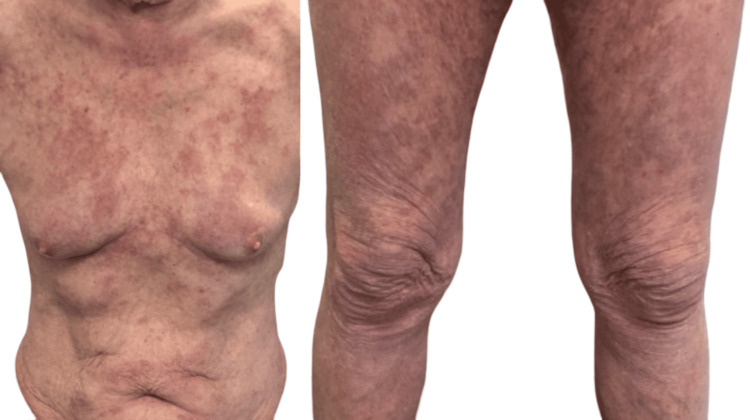
Clinical images of rash showing significant improvement after surgical wedge resection of lung nodule.

The patient underwent surgical wedge resection of the left lower lobe nodule in February 2025. Histopathology confirmed non-small cell lung carcinoma, squamous cell subtype, pathologically staged as T1c N0 M0. Following surgical resection, the patient experienced a rapid and significant improvement in his skin rash, noted at review in the dermatology clinic in March 2025 (Figure 4).

## Discussion

IGD is most commonly associated with autoimmune conditions such as rheumatoid arthritis, SLE, and inflammatory bowel disease, as well as adverse drug reactions [1,2]. Its presentation as a paraneoplastic syndrome, however, is exceedingly rare and remains poorly understood [3,4,7]. This case highlights a likely paraneoplastic IGD associated with a PET-avid cavitating lung nodule, emphasising the need for heightened clinical suspicion in patients with persistent granulomatous dermatoses and systemic symptoms [3,4].

Pathophysiology of paraneoplastic IGD

The precise mechanisms underlying paraneoplastic IGD are not well defined. It is thought to involve immune dysregulation triggered by underlying malignancy, with tumour antigens provoking cross-reactive immune responses against skin components, leading to granulomatous inflammation [7]. In this patient, the presence of a squamous cell carcinoma of the lung could have driven chronic systemic inflammation through cytokine release or antigenic mimicry, precipitating IGD [4]. Persistent elevation of ESR and CRP supports this inflammatory state [3].

Hypercalcaemia, as seen in this case, is a recognised paraneoplastic feature of squamous cell lung carcinoma and is typically mediated by parathyroid hormone-related peptide (PTHrP) [5]. This biochemical disturbance may contribute to systemic inflammation and could potentially exacerbate cutaneous manifestations.

Diagnostic complexity and pitfalls

Diagnosing IGD can be challenging because its clinical and histopathological features overlap with other granulomatous dermatoses [1]. In this case, the initial diagnosis of PRP was made based on clinical presentation [6]. PRP typically manifests as widespread erythematous plaques with follicular hyperkeratosis and islands of sparing, but histology reveals psoriasiform hyperplasia with alternating orthokeratosis and parakeratosis, findings absent in our patient [6].

A subsequent punch biopsy suggested granuloma annulare [5], which is characterised by necrobiotic collagen surrounded by palisading histiocytes and mucin deposition [5]. IGD, in contrast, shows mid-dermal non-caseating granulomas with multinucleate giant cells, a preserved Grenz zone, and no necrobiosis or mucin [1,3]. In this case, the superficial biopsy likely missed the deeper dermal changes, leading to misdiagnosis. This underscores the importance of performing deep incisional biopsies in persistent or atypical lesions [5].

Therapeutic and prognostic considerations

Paraneoplastic IGD is often refractory to conventional therapies such as topical corticosteroids or systemic immunosuppressants [4]. The most effective treatment lies in addressing the underlying malignancy. In our case, complete resection of the lung tumour led to rapid and substantial improvement in the skin eruption, confirming the paraneoplastic nature of the IGD [3,4]. This is consistent with previous reports of paraneoplastic dermatoses resolving after successful cancer treatment [7].

While biologic agents have been used in refractory IGD cases associated with autoimmune disease, their role in paraneoplastic IGD is uncertain and warrants further study [7]. For now, the primary therapeutic strategy should focus on identifying and treating the associated malignancy [3,4].

Clinical implications

This case demonstrates the value of a multidisciplinary diagnostic approach when managing complex skin disorders with systemic manifestations. Persistent, unexplained granulomatous eruptions should prompt clinicians to investigate for systemic disease, including malignancy [3,4], particularly when accompanied by constitutional symptoms or abnormal laboratory findings. Early identification and management of an underlying neoplasm can improve both dermatologic and overall patient outcomes [3,4,7,9].

Key clinical message

IGD is a rare dermatosis that may present as a paraneoplastic syndrome, where adequate deep tissue biopsy is essential to distinguish it from mimics such as granuloma annulare. Persistent granulomatous skin eruptions should prompt systemic evaluation for malignancy, and resolution following cancer treatment supports a paraneoplastic link.

## Conclusions

This case adds to the limited but growing body of evidence supporting the association between IGD and underlying malignancy. It highlights the importance of a comprehensive, multidisciplinary approach when evaluating persistent granulomatous skin diseases, particularly when accompanied by systemic symptoms. Early recognition and treatment of the underlying cause, especially neoplastic processes, are critical to improving patient outcomes. Further research is needed to better understand the mechanisms linking IGD with malignancy and to explore effective management strategies for paraneoplastic IGD.
